# Request for public discussion and ballot to add rules on paratypes to the SeqCode and amend recommendations on name formation

**DOI:** 10.1093/ismeco/ycaf238

**Published:** 2026-01-10

**Authors:** William B Whitman, Iain Sutcliffe, Konstantinos T Konstantinidis, Luis M Rodriguez-R

**Affiliations:** Department of Microbiology, University of Georgia, Athens, GA 30602-2605, United States; School of Geography and Natural Sciences, Faculty of Science and Environment, Northumbria University, Newcastle upon Tyne, NE1 8ST, United Kingdom; School of Civil and Environmental Engineering and School of Biological Sciences, Georgia Institute of Technology, Atlanta, GA 30332-0512, United States; Department of Chemistry and Biosciences, Aalborg University, 9220 East Aalborg, Denmark

**Keywords:** SeqCode, paratypes, amendments

The “Code of Nomenclature of Prokaryotes Described from Sequence Data” (the SeqCode) was conceived and introduced to address the need to validly publish names for taxa known from sequence data [1, 2]. While the SeqCode provides a valuable mechanism for validly publishing names for taxa known from sequence data, a concern is that it insufficiently acknowledges the importance of cultivated strains in the description of species and subspecies. While genome sequences allow the unambiguous identification of species, examination of cultures in the laboratory is often critical for understanding the properties of species and greatly enhances their descriptions. Therefore, an amendment to the SeqCode is proposed to provide formal recognition of strains that add to the species or subspecies descriptions as paratypes. Paratypes may be denoted by the original authors of the description or subsequent authors and must be deposited in at least one publicly accessible culture collection. Crucially, a paratype does not replace the type, which is a DNA sequence. In addition, amendments are proposed to reduce the recommended number of unique letters in a name from three to two and add to Recommendation 28 regarding citation of the effective publication of a new taxon.

## Paratype amendments

Whilst genome sequences can be used to unambiguously identify and classify taxa and hence to underpin their naming, they are not the full characterization of an organism. Even though many physiological and metabolic properties of a microorganism can be predicted from its genome sequence [3], the functions of many genes in the genome remain unknown, and interactions between gene products are poorly understood. As a consequence, the predictions of an organism’s properties from the genome may often require experimental verification. The availability of live cultures enables experimental verification of properties inferred from a genome sequence as well as discovery of additional novel properties.

The importance of strains is acknowledged in the SeqCode by Recommendation 19, which highlights that “When a strain belonging to a taxon named under the SeqCode is isolated, a reference strain should be designated and submitted to two culture collections in different countries” [1]. However, such strains are not otherwise indicated in the SeqCode nomenclature and are difficult to associate with the type genomes.

In zoology and botany, a paratype is a specimen which helps define the species, but it is not the name-bearing type (holotype) or isotype [4, 5]. It is “alongside” the type but not the type. Paratypes are often included in the original description, and there may be more than one. In view of the need to highlight the importance of strains in microbiology, it is proposed here to strengthen SeqCode Recommendation 19 by introducing new Rules that provide recognition of paratypes. If this proposal is accepted for the SeqCode, paratypes may be denoted by the original or subsequent authors with the intention of providing additional information or resources for the description of a species or subspecies. For the SeqCode, paratypes are defined as isolated strains deposited in at least one international culture collection, which differs somewhat from the practice of the “International Code of Nomenclature of Prokaryotes” (ICNP) where Rule 30 requires deposition of type strains in two collections [6]. For species or subspecies whose type genome sequences are obtained from an isolate, the isolate can serve as a paratype. Alternatively, or in addition, after the original description, authors may designate a strain as a paratype if, in their opinion, the genome sequence of that strain is similar enough to the type sequence to be classified in the same species or subspecies. It is emphasized that a paratype does not replace the type, which is a DNA sequence. When it is desirable to indicate that a strain is a paratype, the superscript “Pt” may be attached to its name. As of 17 November 2025, of the 625 species names validly published under the SeqCode, 151 are associated with strains. Of these, 51 names have been associated with reference strains that meet the standards of the proposed Rule 19, while 45 would also meet the standards of ICNP Rule 30 3(B) (i.e. available in culture collections in two different countries). As part of this proposal, if accepted, the strains listed in Table 1 would be designated as paratypes.

**Table 1:** Species names validly published under the SeqCode with reported strains meeting the paratype designation requirements in the present proposal.

Species name	Authority	Paratype publication (DOI)	Paratype designation	Deposition countries
*Actinotalea subterranea*	Semenova et al., 2022 (priority 2025)	Semenova et al., 2022 (10.3390/microorganisms10020378)	HO-Ch2 = VKM Ac-2850 = KCTC 49656	RU, KR
*Agrobacterium albertimagni*	Salmassi et al., 2002 (priority 2025)	Salmassi et al., 2002 (10.1080/014904502317246165)	AOL15 = ATCC BAA-24	USA
*Agrobacterium bohemicum*	Zahradník et al., 2018 (priority 2025)	Zahradník et al., 2018 (10.1016/j.syapm.2018.01.003)	R90 = CCM 8736 = DSM 104667	CZ, DE
*Agrobacterium fabrum*	Lassalle et al., 2011 (priority 2025)	Robert Dickey in Hamilton, Fall, 1971 (10.1007/BF02145913)	C58 = ATCC 33970 = LMG 287 = CIP 104333 = LMD 85.29 = NCCB 85029 = CGMCC 1.1488 = CFBP 1903 = CFBP 6552	USA, BE, FR, CN
*Amycolatopsis camponoti* ^a^	Zakalyukina et al., 2022 (priority 2023)	Zakalyukina et al., 2022 (10.1007/s10482-022-01716-w)	A 23 = DSM 111725 = VKM 2882 = VKM Ac-2882	DE, RU
*Ciceribacter sichuanensis*	Zhang et al., 2024 (priority 2025)	Zhang et al., 2024 (10.1007/s10482-024-01941-5)	S101 = CGMCC 1.61309 = GDMCC 1.3292 = JCM 35649	CN, JP
*Curtobacterium aetherium*	Mijatović Scouten et al., 2025	Bryan et al., 2019 (10.1038/s41396-019-0474-0)	L6–1 = LMG 33684 = ATCC TSD-458	BE, USA
*Enterovibrio baiacui*	*corrig*. Azevedo et al., 2020 (priority 2023)	Azevedo et al., 2020 (10.1007/s00284-019-01785-7)	A649 = CBAS 716 T = CBRVS P1061T	BR
*Fervidibacter sacchari* ^a^	Nou et al., 2024	Nou et al., 2024 (10.1038/s41467-024-53 784-3)	PD1 = JCM 39283 = DSM 113467	JP, DE
*Marinarcus aquaticus*	*corrig*. Pérez-Cataluña et al., 2018 (priority 2025)	Pérez-Cataluña et al., 2018 (10.3389/fmicb.2018.02077)	W112–28 = CECT 8987	ES
*Mesorhizobium denitrificans*	Siddiqi et al., 2019 (priority 2025)	Siddiqi et al., 2019 (10.1007/s12275-019-8590-0)	LA-28 = KACC 19675 = LMG 30806	KR, BE
*Mesorhizobium xinjiangense*	Meng et al., 2022 (priority 2025)	Meng et al., 2022 (10.1007/s00203-021-02686-9)	lm94 = KCTC 72863 = CCTCC AB2019377	KR, CN
*Mesorhizobium zhangyense*	Xu et al., 2018 (priority 2025)	Xu et al., 2018 (10.1007/s00203-017-1464-0)	23-3-2 = CGMCC 1.15528 = NBRC 11233	CN, JP
*Methanacia filiformis*	Protasov et al., 2023 (priority 2024)	Leadbetter et al., 1998 (10.1007/s002030050574)	RFM-3 = DSM 11501 = JCM 39454	DE, JP
*Methanarmilla boviskoreani*	Protasov et al., 2023 (priority 2024)	Lee et al., 2013 (10.1099/ijs.0.054056-0)	JH1 = DSM 25824 = JCM 18376 = KCTC 4102	DE, JP, KR
*Methanarmilla wolinii*	Protasov et al., 2023 (priority 2024)	Miller et al., 1986 (10.1016/S0723-2020(86)80084-4)	SH = ATCC BAA-1170 = DSM 11976	USA, DE
*Methanimicrococcus hacksteinii* ^a^	Protasov et al., 2023 (priority 2024)	Protasov et al., 2023 (10.3389/fmicb.2023.1281628)	DSM 115570 = JCM 39383	DE, JP
*Methanimicrococcus hongohii* ^a^	Protasov et al., 2023 (priority 2024)	Protasov et al., 2023 (10.3389/fmicb.2023.1281628)	Hf6 = DSM 114388 = JCM 3938	DE, JP
*Methanobaculum cuticulare*	*corrig*. Protasov et al., 2023 (priority 2024)	Leadbetter, Breznak, 1996 (10.1128/aem.62.10.3620-3631.1996)	RFM-1 = DSM 11139 = JCM 39453	DE, JP
*Methanobinarius arboriphilus*	Protasov et al., 2023 (priority 2024)	Zeikus, Henning, 1975 (10.3389/fmicb.2023.1281628)	JCM 13429 = DSM 1125 = DH1	JP, DE
*Methanocatella gottschalkii*	Protasov et al., 2023 (priority 2024)	Miller et al., 1986 (10.1016/S0723-2020(86)80084-4)	HO = ATCC BAA-1169 = DSM 11977	USA, DE
*Methanocatella millerae*	Protasov et al., 2023 (priority 2024)	Rea et al., 2007 (10.1099/ijs.0.63984-0)	ZA-10 = DSM 16643 = JCM 39457 = OCM 820	DE, JP, USA
*Methanocatella oralis*	Protasov et al., 2023 (priority 2024)	Brusa et al., 1987 (10.1111/j.1600-051X.1987.tb02254.x)	ZR = DSM 7256 = JCM 30027	DE, JP
*Methanocatella smithii*	Protasov et al., 2023 (priority 2024)	Smith, 1966 (DOI N/A: Smith P.H. 1966. *Dev. Ind. Microbiol.* 7: 156–161.)	ATCC 35061 = PS = DSM 861	USA, DE
*Methanocatella thaueri*	Protasov et al., 2023 (priority 2024)	Miller, Lin, 2002 (10.1099/00207713-52-3-819)	CW = DSM 11995 = OCM 817	DE, USA
*Methanocatella woesei*	Protasov et al., 2023 (priority 2024)	Miller et al., 1986 (10.1016/S0723-2020(86)80084-4)	GS = DSM 11979 = OCM 815 = JCM 39456	DE, USA, JP
*Methanoflexus curvatus*	Protasov et al., 2023 (priority 2024)	Leadbetter, Breznak, 1996 (10.1128/aem.62.10.3620-3631.1996)	RFM-2 = DSM 11111 = JCM 39452	DE, JP
*Methanolapillus africanus* ^a^	Protasov et al., 2023 (priority 2024)	Protasov et al., 2023 (10.3389/fmicb.2023.1281628)	DSM 115569 = JCM 39381	DE, JP
*Methanolapillus ohkumae* ^a^	Protasov et al., 2023 (priority 2024)	Protasov et al., 2023 (10.3389/fmicb.2023.1281628)	DSM 114424 = JCM 39382	DE, JP
*Methanorbis furvi*	Protasov et al., 2023 (priority 2024)	Protasov et al., 2023 (10.3389/fmicb.2023.1281628)	Ag1 = DSM 115764	DE
*Methanorbis rubei*	Protasov et al., 2023 (priority 2024)	Protasov et al., 2023 (10.3389/fmicb.2023.1281628)	Cs1 = DSM 115765	DE
*Mycoplana subbaraonis*	(*ex* Ramana et al., 2013) Hördt et al., 2020 (priority 2025)	Ramana et al., 2013 (10.1099/ijs.0.041442-0)	JC85 = DSM 24765 = KCTC 23614	DE, KR
*Neorhizobium lilii*	*corrig*. Liu et al., 2020 (priority 2025)	Liu et al., 2020 (10.1007/s00203-019-01774-1)	24NR = ACCC 61588 = JCM 33731	CN, JP
*Pampinifervens diazotrophicum*	Palmer et al., 2025	Palmer et al., 2025 (10.1016/j.syapm.2025.126644)	T-2 = JCM 35475 = DSM 116324	JP, DE
*Pampinifervens florentissimum*	Palmer et al., 2025	Palmer et al., 2025 (10.1016/j.syapm.2025.126644)	T-8 = JCM 33569 = CGMCC 1.5214	JP, CN
*Pararhizobium mangrovi*	Li et al., 2021 (priority 2025)	Li et al., 2021 (10.1007/s00284-021-02434-8)	BGMRC 6574 = KCTC 72636 = CGMCC 1.16783	KR, CN
*Peteryoungia desertarenae*	Rahi et al., 2021 (priority 2025)	Rahi et al., 2021 (10.1007/s00203-021-02349-9)	ADMK78 = MCC 3400 = KACC 21383 = JCM 33657	IN, KR, JP
*Phyllobacterium pellucidum* ^a^	Park et al., 2021 (priority 2025)	Park et al., 2021 (10.1007/s00203-021-02205-w)	BT25 = KCTC 62765 = NBRC 114381	KR, JP
*Pseudaminobacter pseudosoli*	corrig. Zhang et al., 2022 (priority 2025)	Zhang et al., 2022 (10.1007/s00284-021-02717-0)	HC19 = KCTC 82870	KR
*Regnicoccus antarcticus*	(*ex* Coutinho et al., 2016; Walter et al., 2017) Ernster, Rodriguez-R, 2025	Waterbury et al., 1986 (DOI N/A: *Can. Bull. Fish. Aquat. Sci.* 214**:** 71–120.)	WH 5701 = SYN = CCMP 1333 = NEPCC 539	USA, CA
*Rhizobium glycinendophyticum*	Wang et al., 2020 (priority 2025)	Wang et al., 2020 (10.1007/s10482-019-01324-1)	CL12 = GDMCC 1.1597 = KACC 21281	CN, KR
*Rhizobium indicum*	Rahi et al., 2020 (priority 2025)	Rahi et al., 2020 (10.1016/j.syapm.2020.126127)	JKLM 12A2 = JCM 33658 = KACC 21380 = MCC 3961	JP, KR, IN
*Rhizobium oryzihabitans*	Zhao et al., 2020 (priority 2025)	Zhao et al., 2020 (10.3390/microorganisms8040608)	M15 = ACCC 60121 = JCM 32903	CN, JP
*Rhizobium quercicola*	Wang et al., 2022 (priority 2025)	Wang et al., 2022 (10.1007/s00203-022-03188-y)	DKSPLA3 = CFCC 16707 = KCTC 82843	CN, KR
*Roseiconus* *lacunae*^a^	Kumar et al., 2021 (priority 2023)	Kumar et al., 2021 (10.1007/s00203-020-02078-5)	JC635 = KCTC 72164 = NBRC 113875	KR, JP
*Roseiconus nitratireducens* ^a^	Kumar et al., 2021 (priority 2023)	Kumar et al., 2021 (10.1007/s00203-020-02078-5)	JC645 = KCTC 72174 = NBRC 113879	KR, JP
*Shinella sumterensis*	Arya et al., 2022 (priority 2025)	Arya et al., 2022 (10.1128/aem.01868-21)	MEC087 = DSM 27294 = ATCC TSD-106	DE, USA
*Taurinivorans muris*	Ye et al., 2023 (priority 2024)	Ye et al., 2023 (10.1038/s41467-023-41 008-z)	LT0009 = DSM 111569 = JCM 34262	DE, JP
*Thalassovivens spotae*	Lanclos et al., 2025	Lanclos et al., 2025 (10.1093/ismeco/ycaf068)	US3C007 = ATCC TSD-433 = NCMA B160 = DSM 119208	USA, DE
*Vibrio fluminensis*	de Azevedo et al., 2022 (priority 2023)	de Azevedo et al., 2022 (10.1007/s00203-022-03266-1)	A621 = CBAS 741 = CAIM 1945 = CCMR 150	BR, MX
*Vibrio tetraodonis*	Azevedo et al., 2021 (priority 2023)	Azevedo et al., 2021 (10.1007/s00203-020-02019-2)	A511 = CBAS 712 = CAIM 1939	BR, MX

The species name and nomenclatural authority are given as currently standing under the SeqCode. The paratype publication and designation are indicated as they would apply under the current proposal. The different countries in which paratype designations have been established are also indicated as ISO 3166–1 two-letter country codes.

a Indicates names that have also been validly published under the ICNP.

Implementation of the rules for paratypes requires amendments in five sections in the SeqCode: Rule 18, Rule 19, Recommendation 19, Recommendation 28, and chapter 4. The proposed amendments immediately follow the original text below. Note that, in addition to the new Recommendation 28c relating to paratypes, another new Recommendation (28b) has been added in order to address the practicalities of citing the effective publication of names upon validation via the SeqCode registry when based on earlier published work that cannot itself serve as an effective publication.

## Original text: type of a species or subspecies

### Rule 18a

The type of a species or subspecies is a designated DNA sequence that is compliant with the minimum standards designated by the SeqCode Committee for genome, metagenome-assembled genome, or single-amplified genome sequences. The sequence must be available in the International Nucleotide Sequence Database Collaboration (INSDC). Upon recommendations of the SeqCode Committee or subcommittees on the taxonomy of specific groups, the SeqCode Committee may approve other minimal standards as suitable types for specific groups.

### Rule 18b

The type of a species or subspecies must allow the unambiguous identification of the taxon. Names based on types that later prove to be ambiguous are not legitimate unless a neotype is proposed.

### Rule 18c

If the type of a name is lost or demonstrated to be ambiguous, a neotype sequence may be proposed to the SeqCode Reconciliation Commission. If approved, the SeqCode Registry will be amended to reflect the new type.

## Suggested amendments of Rule 18 (new text underlined)

### Rule 18a

The type of a species or subspecies is a designated DNA sequence that is compliant with the minimum standards designated by the SeqCode Committee for genome, metagenome-assembled genome, or single-amplified genome sequences. The sequence must be available in the INSDC. Upon recommendations of the SeqCode Committee or subcommittees on the taxonomy of specific groups, the SeqCode Committee may approve other minimal standards as suitable types for specific groups.

### Rule 18b

The type of a species or subspecies must allow the unambiguous identification of the taxon. Names based on types that later prove to be ambiguous are not legitimate unless a neotype is proposed.

### Rule 18c

If the type of a name is lost or demonstrated to be ambiguous, a neotype sequence may be proposed to the SeqCode Reconciliation Commission. If approved, the SeqCode Registry will be amended to reflect the new type.

### Rule 18d

Unless designated under the rules of this code, a reference DNA  sequence  is  not  a  type  but  a  sequence  used in comparative  studies. A reference sequence has no standing in nomenclature.

## Original text: Rule 19 (proposed to become Rule 18d)

Unless designated under the rules of this code, a reference DNA sequence is not a type but a sequence used for comparative studies. A reference sequence has no standing in nomenclature.

## Suggested amendments of Rule 19 (new text underlined)

### Rule 19a

When a strain belonging to a taxon named under the  SeqCode is   isolated, it may be designated as a **paratype**, i.e. a specimen that  provides additional material for the description of a species or su  bspecies. To  qualify  as a  paratype, a  viable culture of that strain   must be  deposited  in at  least  one publicly accessible culture co  llection  from  which  subcultures  must  be  available.  A  paratype  does not replace a sequence as the type. A strain may be designa  ted a paratype by the original or subsequent authors.

### Rule 19b

If  the  type  sequence  is  obtained  from  an isolated strain, then  that  strain  is  a  paratype  if it is deposited as designated in Rule  19a.

### Rule 19c

More  than  one  paratype  may  be  designated  for  a  species  or  subspecies.

## Original text: Recommendation 19

When a strain belonging to a taxon named under the SeqCode is isolated, a reference strain should be designated and submitted to two culture collections in different countries. Reference strains have no standing in nomenclature.

## Suggested amendment of Recommendation 19

This recommendation is deleted.

## Original text: proposal and subsequent citation of the name of a new taxon

### Recommendation 28

The effective publication should be cited with the name of a previously proposed taxon. Correct citation of a name enables the date of publication, the description, and the circumscription of the taxon to be found. For names published under the SeqCode, the validly published name and date of valid publication should be determined from the SeqCode Registry.

## Suggested amendments of Recommendation 28 (new text underlined)

### Recommendation 28a

The effective publication should be cited with the name of a previously proposed taxon. Correct citation of a name enables the date of publication, the description, and the circumscription of the taxon to be found. For names published under the SeqCode, the validly published name and date of valid publication should be determined from the SeqCode Registry.

### Recommendation 28b

If a publication proposing a name fails to meet the criteria for  valid publication, the publication should be cited in parentheses  preceding the effective publication and following the term “*ex*”  upon valid publication of the name.

### Recommendation 28c

The first  publication  reporting or establishing an isolated strain  designated  as a  paratype  under  Rule  19a should be cited after  the effective publication, following the term “paratype” or “Pt.”

## Original text: chapter 4. Recommendations for authors and publishers

When it is desirable to distinguish the nature of the type of a name, the following convention is recommended. When the type for a species or subspecies is determined by the ICNP, the superscript “T” will be used immediately following the name or strain identifier. If the type is determined by the SeqCode, the superscript “Ts” or “TS” will be used. When the type is a taxon at the rank of genus or higher, the superscript is determined by the nature of the type of the species. If superscripts are not possible, they may be replaced by the symbols in parentheses, i.e. (T), (Ts) or (TS).

For the purpose of identification in the text, names of taxa at all ranks should be italicized.

### Suggested amendments of chapter 4 (new text underlined)

When it is desirable to distinguish the nature of the type of a name, the following convention is recommended. When the type for a species or subspecies is determined by the ICNP, the superscript “T” will be used immediately following the name or strain identifier. If the type is determined by the SeqCode, the superscript “Ts” or “TS” will be used. When the type is a taxon at the rank of genus or higher, the superscript is determined by the nature of the type of the species. If a paratype is to  be  designated, the superscript “Pt” or “PT” will  be used. If superscripts are not possible, they may be replaced by  the symbols in parentheses, i.e. (T), (Ts) or (TS), and (Pt) or (PT).

For the purpose of identification in the text, names of taxa at all ranks should be italicized.

### Recommendation for the formation of the names amendment

Recommendation 9.2 encourages authors to create names with three or more different letters from preexisting names. The goal was to make names more recognizable and easier to distinguish. However, when this recommendation was implemented, it was found that it was often difficult to create satisfying names with three differences from preexisting names and that many names differing by only two letters were easily distinguished. The specification of a number of different letters in the SeqCode is interpreted to be identical to the Levenshtein distance, which corresponds to the minimum number of individual edits required to transform a word into another. Individual edits are single-letter insertions, deletions, or substitutions. Examples of names with a Levenshtein distance of three include: *Allotabrizicola* with *Neotabrizicola*; *Houyiibacterium* with *Hominibacterium* and *Fluviibacterium*; *Xiheimicrobium* with *Luteimicrobium* and *Roseimicrobium*; *Tepidihabitans* with *Calidihabitans*, *Tropicihabitans*, and *Terrihabitans*; and *Pelagibacter* with 18 names including *Clavibacter*, *Pilosibacter*, and *Algibacter*. Whilst some pairs of names with a Levenshtein distance of two might be easily confused (such as: *Lacustribacter* and *Lustribacter*; *Allotabrizicola* and *Aliitabrizicola*; *Freyriarchaeum* and *Freyarchaeum*; and *Hahnella* and *Hallella*), other pairs with this distance differ sufficiently to avoid confusion (e.g. *Hahnella* and *Taonella*; *Benthobacter* and *Xanthobacter*). Indeed, this limit is both practical and objective and affects fewer names overall. In all, only 20%, 30%, and 11% of names validly published under SeqCode, ICNP, and ICNafp, respectively, have a distance of two or less to other validly published names. For these reasons, it is proposed to change the recommendation to create names with two or more differences.

### Original text: Recommendation 9

To form new prokaryotic names, authors are advised as follows:

1. Names should differ by at least three characters from existing names of genera or species within the same genus.

### Suggested amendment: Recommendation 9

To form new prokaryotic names, authors are advised as follows:

1. Names should differ by at least two characters from existing names of genera or species within the same genus.

### Balloting on amendments

As specified in Article 11 of the SeqCode Statutes [7], amendments to the SeqCode must be proposed by publication of a peer-reviewed article explaining the rationale for the proposed changes and clearly stating the new wording. Following publication, the Chair of the SeqCode Legislative Commission will initiate community discussion of the proposal for a period of not less than 3 months and not longer than 6 months. Following the authors’ opportunity to respond, their response and the public discussion will be compiled by the Secretary of the Legislative Commission and communicated to the Secretary of the Executive Board within 1 month. The Executive Board will then disseminate the materials prepared by the Legislative Commission and arrange for a ballot of the SeqCode Community.

### In summary

We welcome public discussion of these proposed amendments to the SeqCode via the SeqCode Public Channel (http://seqco.de/connect), followed by a ballot of the SeqCode Community. Those interested in these issues are invited to join the SeqCode Community via the SeqCode Community Signup form (https://seqco.de/join).

## Data Availability

Data sharing is not applicable to this article, as no datasets were generated or analyzed during the current study.
